# *Lawsonia intracellularis* LI0666 is a new EPIYA effector exported by the *Yersinia enterocolitica* type III secretion system

**DOI:** 10.1186/s13567-022-01054-9

**Published:** 2022-06-04

**Authors:** Cang Chen, Yimin Dai, Yingying Yang, Zihe Zhu, Qinghua Zhang, Xuejiao An, Fenju Lai

**Affiliations:** 1 College of Bioscience and Bioengineering, Jiangxi Key Laboratory for Conservation and Utilization of Fungal Resources, Jiangxi Agricultural University, Nanchang, 330045 China; 2 College of Animal Science and Technology, Jiangxi Agricultural University, Nanchang, 330045 China; 3 Collaborative Innovation Center of Postharvest Key Technology and Quality Safety of Fruits and Vegetables in Jiangxi Province, Nanchang, Jiangxi China

**Keywords:** *Lawsonia intracellularis*, EPIYA effectors, Tyrosine phosphorylation, *Yersinia* T3SS

## Abstract

*Lawsonia intracellularis* is the causative agent of proliferative enteropathy. While it harbors genes encoding the entire apparatus required for the type III secretion system (T3SS) and the expression of some of these components has been detected during experimental infection, the identification of *L. intracellularis* T3SS substrates (effector proteins) has been hampered. The *Yersinia* T3SS and yeast growth inhibition assays are two important heterologous systems used for the characterization of effector proteins. Bacterial EPIYA effectors are a distinct class of bacterial effectors defined by the presence of EPIYA or the EPIYA-related motif. When delivered into host cells via a T3SS or type IV secretion system, these effectors undergo tyrosine phosphorylation of the EPIYA motif, which enables them to manipulate host cell signaling by promiscuously interacting with multiple SH2 domain-containing proteins. A previous study showed that *L. intracellularis* LI0666 contains two EPIYA motifs and speculated that this protein could be a T3SS effector. In this study, we show that LI0666 is secreted by *Yersinia* in a T3SS-dependent manner and inhibits yeast growth. LI0666 is phosphorylated at tyrosine residues in porcine intestinal epithelial cells and in human epithelial cells. Like the archetypal EPIYA effector CagA, the EPIYA-containing region is not required for LI0666 association with yeast and mammalian cell membranes. Our results indicate that LI0666 is an authentic bacterial EPIYA effector. Identification of the tyrosine kinases that are responsible for LI0666 phosphorylation and the SH2 domain-containing host proteins that LI0666 interacts with will help to explore the molecular mechanisms of LI0666 in disease development.

## Introduction

*Lawsonia intracellularis* is a Gram-negative, obligate, intracellular bacterial pathogen that infects a wide range of animals, mainly pigs and horses, and causes the contagious disease known as proliferative enteropathy [1–3]. Although *L. intracellularis* was successfully propagated in rat small intestinal cells in 1993 [4], growth of this bacteria in axenic (cell-free) media has not been achieved. Despite decades of research, the pathogenesis and virulence factors of this organism have not been well-characterized. The sequence of the *L. intracellularis* genome indicates that it may possess a type III secretion system (T3SS), which could assist the bacterium during cell invasion and evasion of the host’s immune system and induce cellular proliferation [5]. While the expression of some components of this putative T3SS has been detected during experimental infection, the microaerophilic obligate intracellular lifestyle and the genetic dissimilarity between *L. intracellularis* and other enteric pathogens have hampered the identification of potential T3SS substrates (effector proteins) [5]. Intracellular bacteria typically require the activity of many effector proteins to remodel the host environment and establish a replicative niche, as exemplified by *Chlamydia* spp [6], which suggests that effector proteins play a significant role in the pathogenesis of *L. intracellularis*. Given the challenges of culturing *L. intracellularis* under laboratory conditions, currently the most feasible method for effector protein identification is the use of a surrogate system.

The Ysc-Yop T3SS from *Yersinia* was the first T3SS to be characterized, and is considered the archetype [7, 8]. The Yop effector proteins (YopH, YopO/YpkA, YopP/YopJ, YopE, YopM, YopT) and the secretion apparatus used to export them from the bacteria into eukaryotic cells—the injectisome—are all encoded by a 70-kb virulence plasmid called pYV in *Y. enterocolitica* [9]. Secretion and translocation of the Yop effectors are normally triggered by contact with a eukaryotic cell. However, secretion can be artificially induced by chelating Ca^2+^ ions, which leads to a massive release of Yops into the culture supernatant [10]. The coding sequence for a secretion signal is located at the 5′ end of each *yop* gene, and the first 15 amino acids of YopE constitute the N-terminal secretion signal [11]. The T3SS needle protein YscF is indispensable for effector protein translocation into host cells [12]. The *Yersinia* T3SS is a genetically tractable heterologous system that has been successfully employed to identify novel T3SS substrates from genetically intractable strains [13].

The budding yeast, *Saccharomyces cerevisiae*, is another important heterologous system used for the screening and functional characterization of effector proteins in a eukaryotic environment, which has been used to study over 100 effectors [14]. Yeast growth inhibition is a sensitive and specific indicator of the activity of effector proteins that perturb conserved cellular processes and has been extensively used as a first step in the search for effector function [3, 14]. Recently, we reported that LI1035, the first putative *L. intracellularis* effector, inhibits yeast growth [15].

Bacterial EPIYA effectors are a distinct class of bacterial effectors defined by the presence of the Glu-Pro-Ile-Tyr-Ala sequence (EPIYA motif) or an EPIYA-related motif, many of which have diverged from the original sequence through multiple duplications [16]. When delivered into host cells via a T3SS or type IV secretion system (T4SS), these effectors undergo tyrosine phosphorylation at the EPIYA motif, which enables them to manipulate host cell signaling by promiscuously interacting with multiple SH2 domain-containing proteins [16]. Since the discovery of the archetypal EPIYA effector, *Helicobacter pylori* CagA, and recognition of the crucial roles that these effectors play in disease manifestations during pathogenic bacterial infection, several additional bacterial EPIYA effectors have been identified. To date, ten EPIYA-motif containing effectors have been identified in six pathogens: *H. pylori* CagA, *Anaplasma phagocytophilum* AnkA, enteropathogenic *Escherichia coli* Tir, *Citrobacter rodentium* Tir, *Chlamydia trachomatis* Tarp, *Haemophilus ducreyi* LspA1 and LspA2, and *Bartonella henselae* BepD, BepE, and BepF [16, 17]. An earlier study showed that CagA translocates into gastric epithelial cells and localizes to the inner surface of the plasma membrane, where it undergoes tyrosine phosphorylation of the EPIYA motif [18]. Further research showed that the EPIYA-containing region of CagA is not required for its membrane association [19, 20].

**Figure 1:**
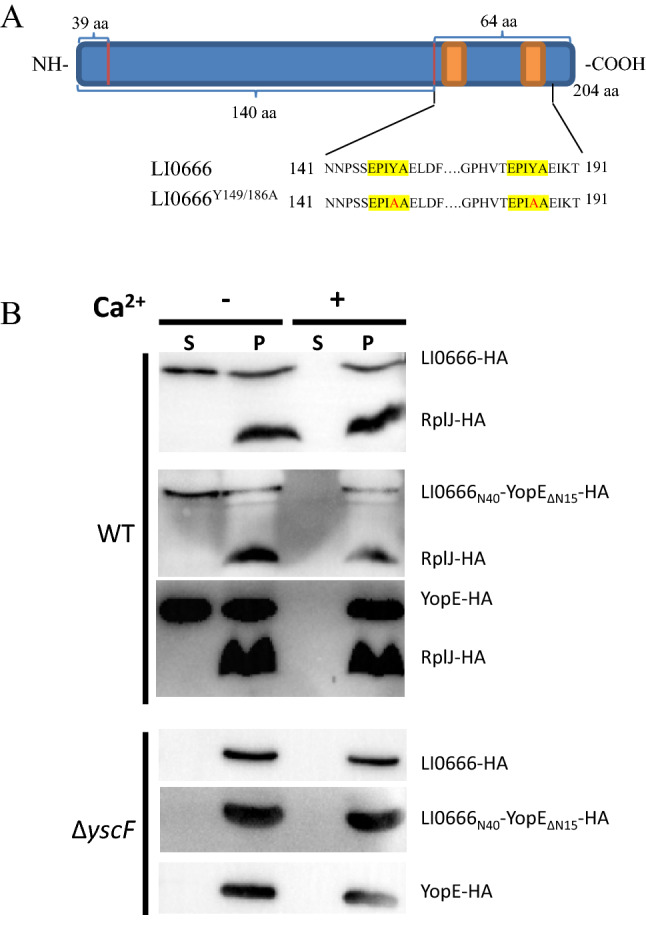
**LI0666 is a substrate of the *Yersinia* type 3 secretion system.** **A** Schematic diagram of LI0666. The EPIYA motif is highlighted in yellow. **B** T3SS-competent *Y. enterocolitica* MRS40_∆YopHOPEM_ expressing HA-tagged proteins (LI0666, LI0666_N40_-YopE_∆N15_, RplJ, YopE) and T3SS-null *Y. enterocolitica* MRS40_∆yscF_ expressing HA-tagged proteins (LI0666, LI0666_N40_-YopE_∆N15_, YopE) were cultivated in BHI under T3SS-repressive (+ Ca^2+^), or -inductive (−Ca^2+^) conditions. Bacteria were grown for 4 h at 37 °C in the presence of 0.2% L-arabinose to induce T3SS and transgene expression. Equal amounts of *Y. enterocolitica* MRS40_∆YopHOPEM_ cultures expressing HA-LI0666 and HA-RplJ, *Y. enterocolitica* MRS40_∆yscF_ cultures expressing HA-LI0666 and HA-YopE, and *Y. enterocolitica* MRS40_∆YopHOPEM_ cultures expressing HA-YopE were centrifuged to separate the cell-free culture supernatants (CS) from the whole-cell pellets (WC), and material corresponding to 0.10 OD_620_ per mL of the original cultures for CS and 0.02 OD_620_ per mL for WC was resolved on 12% (w/v) polyacrylamide gels. Specific proteins were detected by immunoblot with anti-HA followed by visualization with alkaline phosphatase–conjugated secondary antibodies and development with ECL.

Recently, using hidden Markov models, Xu et al. showed that EPIYA-containing proteins are significantly overrepresented in intracellular bacteria, extracellular bacteria with T3SS and T4SS, and intracellular protozoan parasites [17]. Analysis of the PHE/MN1-00 sequence using HMM identified 20 *L. intracellularis* proteins that contain the EPIYA motif. Three hypothetical proteins (LI0041, LI0666, and LIC053) contain two copies of the EPIYA motif. The two copies of the EPIYA motif located in LI0666 (EPIYAEIKT Y-149 and EPIYAEIKT Y-186) are similar to the SH2-domain-binding R4 motif in CagA and the Tir motif in Tir (Figure 1A) [17]. LI0666 was predicted to be an extracellular or outer membrane protein in *L. intracellularis* using CELLO v.2.5 [17], and to be a T3SS effector using the EffectiveT3 program [21], which prompted us to explore it in more detail [17].

Here we show that LI0666 is exported by *Yersinia* in a heterologous expression assay, and its expression inhibits yeast growth, both of which indicate that LI0666 is a bacterial EPIYA effector. LI0666 is phosphorylated at the tyrosine residues within its EPIYA motifs in mammalian cells. Although LI0666 localizes to yeast and mammalian cell membranes, similar to CagA, the EPIYA-containing region is not required for membrane association. Identification of the tyrosine kinases that are responsible for LI0666 phosphorylation and the SH2 domain–containing host proteins that LI0666 interacts with will help to explore the molecular mechanisms of LI0666 in disease development.

## Materials and methods

### Strains and cell lines

The *E. coli* strain DH5α was used to construct and preserve clonal plasmids and was grown in Luria–Bertani (LB) broth. *Y. enterocolitica* MRS40_ΔyopHOPEM_ (lacking the *Yersinia* Yop T3SS effectors YopH, YopO, YopP, YopE, and YopM, but T3S-proficient) and T3SS-deficient *Y. enterocolitica* MRS40_ΔyspF_ were routinely grown in brain heart infusion (BHI; TransGen Biotech, Beijing, China) agar, and 200 µg mL^−1^ ampicillin was added to select expression vectors.

The *S. cerevisiae* strains W303-1A (MATa *ade2-1 ura3-1 his3-11*,*15 trp1-1 leu2-3*,*112 can1-100*) and BY4741 (MATa *his3∆1 leu2∆0 met15∆0 ura3∆0*) were grown at 30°C in yeast-peptone-dextrose (YPD) (1% yeast extract, 2% peptone, 2% glucose) broth or agar (2%) or in selective synthetic complete (SC) medium lacking uracil (SC-Ura) to maintain the plasmid and were supplemented with 2% glucose (SCD), or 2% galactose and 1% raffinose (SCG), as carbon sources. The components for the media were purchased from HiMedia and Difco.

The human embryonic kidney (HEK) cell line 293T, human cervical epithelial cell line HeLa, and porcine intestinal epithelial cell line IPEC-J2 (Guangzhou Jennio Biotech Co., Ltd, Guangzhou, China) were cultured in Dulbecco’s modified Eagle medium (DMEM, Solarbio) supplemented with 10% fetal bovine serum (FBS, Gibco). Cells were maintained at 37 °C in a humidified atmosphere containing 5% CO_2_.

### Plasmid construction

The plasmids and primers (synthesized by Sangon Biotech (Shanghai) Co., Ltd., China) used in this study are described in Tables 1 and 2, respectively. The *YopE* gene (RefSeq: NZ_CP009711.1) from *Y. pseudotuberculosis* with the coding sequence for a C-terminal HA epitope tag fragment was synthesized and cloned into the *Eco*RI/*Sal*I sites of the pBAD24 vector under the control of an araBAD promoter. An *RplJ* gene fragment (RefSeq: NZ_CQAE01000015.1) amplified from genomic DNA of *Y. pseudotuberculosis* MRS40 or an *LI0666* gene fragment (RefSeq: NC_020127.1) amplified from genomic DNA extracted from *L. intracellularis*—positive porcine ileal mucosa was cloned into the *Eco*RI/*Sal*I sites of the pBAD24-YopE-HA vector. The coding sequence for the first 40 amino acids of the *LI0666* gene was amplified from pBAD24-LI0666-HA and inserted into pBAD24-YopE-HA in-frame with the coding sequence for the YopE C-terminus (YopE_Δ15_) by homologous recombination. Plasmids were introduced into *Yersinia* by electroporation.

**Table 1:** **Plasmids used in this study**

Plasmids	Genotype/Description	Source
pRS416-GAL1	GAL1 promoter, 3 × Flag tag, URA3, ampicillin	[15]
pRS416-GAL1-LI0666	GAL1 promoter, 3 × Flag-LI0666, URA3, ampicillin	This study
pRS416-GAL1-LI0666^Y149/186A^	GAL1 promoter, 3 × Flag-LI0666^Y149/186A^, URA3, ampicillin	This study
pYES2URA-RipI	2 µ, GAL1/GAL10, RipI, URA3	[23]
pBAD24-YopE-HA	araC promoter, YopE-HA, ampicillin	This study
pBAD24-RplJ-HA	araC promoter, RplJ-HA, ampicillin	This study
pBAD24-LI0666-HA	araC promoter, LI0666-HA, ampicillin	This study
pBAD24-LI0666_N40_-YopE_∆N15_-HA	araC promoter, LI0666_N40_-YopE_∆N15_-HA, ampicillin	This study
pYES2URA-EGFP	EGFP gene fragment was inserted into pYES2URA under GAL1 promoter	This study
pYES2URA-LI0666-EGFP	LI0666 gene fragment was inserted into pYES2URA-EGFP under GAL1 promoter	This study
pYES2URA-LI0666^Y149A^-EGFP	LI0666 harboring A in place of Y at position 149	This study
pYES2URA-LI0666^Y186A^-EGFP	LI0666 harboring A in place of Y at position 186	This study
pYES2URA-LI0666^Y149/186A^-EGFP	LI0666 harboring A in place of Y at position 149 and 186	This study
pYES2URA-LI0666_△N39_-EGFP	LI0666 harboring deletion for 1–39	This study
pYES2URA-LI0666_△C64_-EGFP	LI0666 harboring deletion for 141–204	This study
pEGFP-C1	CMV, Neomycin, kanamycin, EGFP	Clontech
pEGFP-C1-LI0666^Y149/186A^	LI0666^149/186A^ gene fragment was inserted into pEGFP-C1 in frame with EGFP at the N-terminal	This study
pEGFP-C1-LI0666	LI0666 gene fragment was inserted into pEGFP-C1 in frame with EGFP at the N-terminal	This study
pEGFP-C1-LI0666_△C64_	LI0666 harboring deletion for 141–204 was inserted into pEGFP-C1 in frame with EGFP at the N-terminal	This study

**Table 2:** **Primers used in this study**

Primer	Sequence (restriction enzyme sites are underlined)	Restriction enzyme
LI0666-F	ATTACAAGGATGACGATGACAATGGCGGAGGAGCGGCCGCGATGAAAATTCAATTTAAA	*Not*I
LI0666-R	GCGTGAATGTAAGCGTGACATAACTAATTACATGACTCGAGTTAGTTATTCTTCCCTTT	*Xho*I
LI0666^Y149A^-F	AACCCTTCATCAGAGCCTATTGCTGCAGAACTTGATTTTAC	Fusion PCR
LI0666^Y149A^-R	TGCAGCAATAGGCTCTGATGAAGGGTT	
LI0666^Y186A^-F	CCACATGTAACAGAACCCATTGCTGCTGAAATTAAAACAAC	
LI0666^Y186A^-R	AGCAGCAATGGGTTCTGTTACATGTGG	
pBAD24-YopE-F	CCGGAATTCATGAAAATATCATCATTT	*Eco*RI
pBAD24-YopE-HA-R	CCCAAGCTTAAGCGTAATCTGGTACGTCGTATGGGTAGTCGACCATCAATGACAGTAA	*Hin*dIII *Sal*I
pBAD24-RplJ-F	GTTTTTTTGGGCTAGCAGGAGGAATTCATGGCACTAAATCTTCAA	*Eco*RI
pBAD24-RplJ-HA-R	ATCTGGTACGTCGTATGGGTAGTCGACAGCAGCTTCTTTCTGATC	*Sal*I
pBAD24-LI0666-F	GTTTTTTTGGGCTAGCAGGAGGAATTCATGAAAATTCAATTTAAA	*Eco*RI
pBAD24-LI0666-HA-R	ATCTGGTACGTCGTATGGGTAGTCGACGTTATTCTTCCCTTTATT	*Sal*I
pBAD24-LI0666_N40_-R	GCTAGATCCTGACACAGAGCTCTCTTTTTTAGTAGG	
EGFP-F	ccgGAATTCTGGCAGGTGCTGGTGCTGGTGCTGGAGCAATCCTGGTGAGCAAGGGCGAG	*Eco*RI
EGFP-R	ccgGACTCGAGCTTACTTGTACAGCTCGTC	*Xho*I
LI0666_∆N39_-EGFP-F	CGCAGGATCCAGGGAGTAGAAGAAAATGGT	*Bam*HI
LI0666_∆C64_-EGFP-R	AGCACCTGCCAGAATTCCTTCTGGAATAGGAGGTAACG	*Eco*RI
LI0666-EGFP-F	AAGGTACCTAGGATCCAGATGAAAATTCAATTTAAA	*Bam*HI
LI0666-EGFP-R	AGCACCTGCCAGAATTCCGTTATTCTTCCCTTTATT	*Eco*RI
pEGFP-C1-LI0666-F	ATGGATGAGCTGTACAAGTCCGGACTCAGATCTCGAGCTATGAAAATTCAATTTAAA	*Xho*I
pEGFP-C1-LI0666-R	ATGGCTGATTATGATCAGTTATCTAGATCCGGTGGATCCTTAGTTATTCTTCCCTTT	*Bam*HI
pEGFP-C1-LI0666_∆C64_-R	CGCGGATCCTTATTCTGGAATAGGAGGTAACG	*Bam*HI

The yeast expression vector pRS416-GAL1 has been described previously [15]. Fragments of the LI0666 ORF were PCR-amplified and cloned into the low–copy number vector pRS416 in frame with the coding sequence for an N-terminal 3 × flag tag under the control of a GAL1 promoter to generate the recombinant plasmid pRS416-GAL1-3 × flag-LI0666. Fragments of the LI0666 ORF were amplified by PCR with primer pair LI0666-F/LI0666^Y149A^-R and primer pair LI0666^Y149A^-F/LI0666-R, which were then sewn together by fusion PCR using primers LI0666-F and LI0666-R. The PCR product was used as the template and amplified by PCR with primer pair LI0666-F/LI0666^Y186A^-R and primer pair LI0666^Y186A^-F/LI0666-R, which were sewn together by fusion PCR using primers LI0666-F and LI0666-R. This final PCR product was cloned into pRS416-GAL1 to create the pRS416-GAL1-LI0666^Y149/186A^ plasmid. A full-length EGFP gene was amplified from the pEGFP-C1 plasmid and the amplified DNA products were cloned into pYES2/NTA to construct the pYES2/NTA-EGFP plasmid. DNA fragments encoding full-length LI0666, LI0666^Y149/186A^, or truncated LI0666 genes were cloned into the pYES2/NTA-EGFP and pEGFP-C1 plasmids individually.

### *Yersinia* T3S assays

T3SS-competent *Y. enterocolitica* MRS40_ΔyopHOPEM_ and T3SS-null *Y. enterocolitica* MRS40_ΔyscF_ were used in the T3S assays, which were performed as previously described [22]. *Yersinia* was cultivated in BHI supplemented with either 5 mM EGTA and 20 mM MgCl_2_ (−Ca^2+^) or 5 mM CaCl_2_ (+ Ca^2+^). Gene expression was induced by adding 0.2% L-arabinose to the culture, and proteins in bacterial pellets and culture supernatants were analyzed by immunoblotting with an anti-HA antibody.

### Yeast growth assays

pRS416-GAL1 vectors carrying LI0666 were introduced to yeast strains W303-1A and BY4741. Yeast growth assays were performed as described previously [15]. Briefly, the yeast strains were grown overnight in liquid selective medium containing glucose, then washed and diluted to an OD_600_ of 1. Each strain was then tenfold serially diluted four times and spotted (5 μL) onto repressing (2% glucose) or inducing (2% galactose) solid selective medium. The plates were then incubated at 30 °C for 2–4 days.

### Yeast phosphorylation assays

Yeast strains expressing wild-type or mutated LI0666 were grown in selective SCD-Ura medium at 30 °C until mid-log phase. The cells were then pelleted, washed, and resuspended in selective induction medium (SCG-Ura). After 12 h of induction, the cells were pelleted, and whole-cell protein extracts were prepared. Equal amounts of proteins were fractionated by SDS-PAGE and transferred to PVDF membranes (Millipore, USA). The membranes were probed with either a monoclonal anti-FLAG (Proteintech; Wuhan, China) antibody or a 4G10 antibody (Millipore). The primary antibody was detected using a horseradish peroxidase–conjugated anti-mouse antibody (Millipore), and the blot was developed with ECL chemiluminescent substrate (Proteintech).

### Mammalian cell line growth conditions for protein phosphorylation assay

HEK293T, HeLa, and IPEC-J2 cells were seeded into 10-cm tissue culture plates. Plasmids were transfected into the cells using Lipofectamine^™^ 2000 (Invitrogen) according to the manufacturer’s protocol. The plates were subsequently incubated at 37 °C for 48 h. Cells were washed with ice-cold PBS and then lysed with RIPA lysis buffer (Beyotime Biotechnology, China) containing protease inhibitors and phosphatase inhibitors. Equal volumes of samples were subjected to SDS-PAGE. Proteins were transferred to PVDF membranes and probed with an anti-GFP antibody (Proteintech) and a 4G10 antibody (Millipore).

### Fluorescence microscopy to determine subcellular localization in yeast

Plasmids were transformed into yeast strain W303-1A, which was then grown in SCD-Ura medium at 30°C to exponential phase (OD_600_ ~ 0.8–1.0). Cells were pelleted, washed, and cultured in selective induction medium (2% galactose, 1% raffinose). After 12 h of induction, cells were fixed in 3.7% formaldehyde for 30 min at room temperature and washed twice with PBS containing 1 mg mL^−1^ BSA, and GFP was visualized using conventional laser excitation and filter sets on a confocal laser scanning microscope (Nikon A1R).

### Fluorescence microscopy to determine subcellular localization in mammalian cells

HEK293T cells were seeded at 2.5 × 10^5^ cells/well in a 6-well tissue culture plate. Plasmids were transfected into the HEK293T cells using Lipofectamine^™^ 2000 (Invitrogen) according to the manufacturer’s protocol, and the plates were incubated at 37 °C for 24 h. Next, the transfected cells were grown on coverslips, fixed in 4% paraformaldehyde (PFA, Sigma) for 10 min, and then permeabilized in 0.2% Triton X-100 for 10 min. All steps were performed in 1 × PBS (phosphate buffered saline, Fisher). Coverslips were mounted with DAPI (Sangon Biotech, Shanghai Co., Ltd.), and fluorescence was observed using a fluorescence microscope.

## Results

### Identification of LI0666 as a type III secretion system substrate using the heterologous *Y. enterocolitica* system

LI0666 was predicted to be a T3SS effector by the EffectiveT3 program, which prompted us to investigate it in further detail. Due to the difficulties associated with *L. intracellularis* in vitro cultivation and the lack of a genetic system for this organism, we investigated LI0666 secretion in an alternative host. Hence, we expressed LI0666 in T3S–competent (WT) or –null (∆yscF) *Y. enterocolitica* as an HA-tagged protein under the control of an arabinose-inducible promoter. RplJ (an endogenous ribosomal protein) and YopE (an endogenous T3SS substrate) were used as the negative and positive controls, respectively. Equal amounts of cultured cells expressing LI0666-HA, YopE-HA, or RplJ-HA were centrifuged and fractionated into cell-free culture supernatants and *Yersinia*-containing whole-cell pellets. Immunoblot analysis of WT cultures revealed essentially equal LI0666-specific signals in + Ca^2+^ (repressive) and −Ca^2+^ (inductive) cell pellet fractions, but LI0666 was only detected in the supernatant in T3SS-inductive but not-repressive conditions, as was the positive control YopE. This outcome was not due to bacterial lysis, because the *Yersinia* cytoplasmic protein RplJ was detected only in whole-cell pellet samples and not in the supernatant fractions (Figure 1B). LI0666 was not detected in the supernatant from the ∆yscF strain, nor was the positive control YopE, which also confirmed that secretion of LI0666 was dependent on a functional T3SS in *Yersinia* (Figure 1B).

To further identify the T3S signal of the LI0666 protein, we analyzed secretion of the first 40 amino acids of LI0666 fused to the YopE_ΔN15_. LI0666_N40_-YopE_ΔN15_ was detected in supernatant from WT cultures grown under T3SS-inductive but not -repressive conditions (Figure 1B). LI0666_N40_-YopE_Δ15_ was not detected in the supernatant from the ∆yscF strain, which also confirmed that secretion of LI0666_N40_-YopE_ΔN15_ was dependent on a functional T3SS in *Yersinia* (Figure 1B). These results demonstrate that LI0666 was exported by the *Yersinia* T3SS, and that the secretion signal is located at the N-terminus of LI0666 (aa 1–40).

### Ectopic expression of LI0666 inhibits yeast growth, and this effect is dependent on the tyrosine residues in the EPIYA motifs

**Figure 2:**
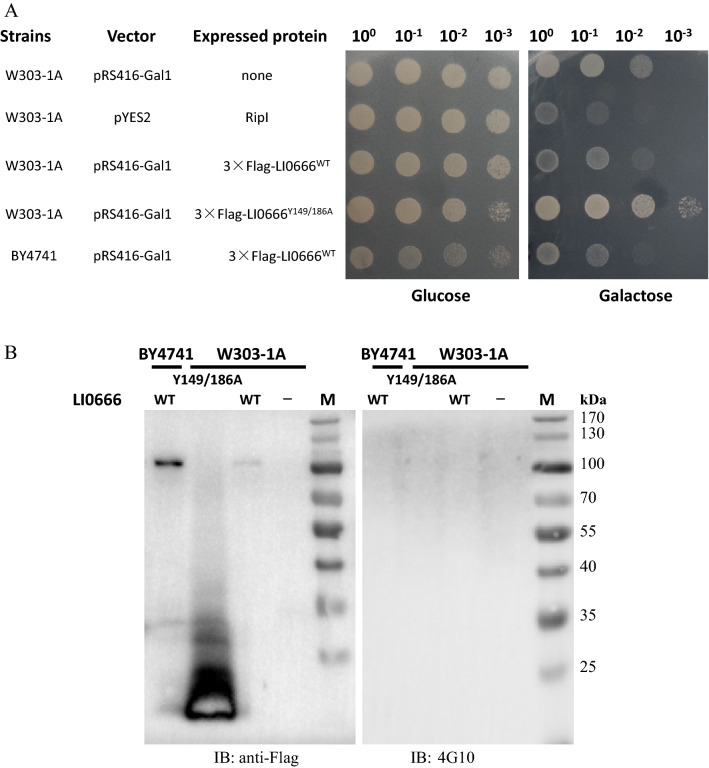
**LI0666 expression modulates yeast growth.** **A** LI0666 expression inhibits yeast growth. W303-1A and BY4741 yeast strains carrying the yeast expression vector pRS416-GAL1, either empty or encoding LI0666 or LI0666^Y149/186A^ with a N-terminal 3 × Flag, were grown overnight in repressing medium (2% glucose). Cultures were then normalized to OD_600_ 1.0, and serial tenfold dilutions were grown at 30 °C for 2 and 3 days in repressing and inducing medium (2% galactose and 1% raffinose), respectively. W303-1A yeast carrying the yeast expression vector pYES2/NT-RipI was used as the positive control. **B** Induction of expression was verified by Western blotting using an anti-Flag antibody and an anti-phosphotyrosine antibody (4G10) for 3 × Flag-LI0666 and 3 × Flag-LI0666^Y149/186A^.

To determine whether LI0666 perturbs cellular functions and inhibits yeast growth, Flag-tagged LI0666 was expressed from an inducible promoter in *S. cerevisiae* strain W303-1A. Under inducing conditions, LI0666 caused severe growth inhibition, comparable to that of the positive control, RipI, a virulent phytopathogenic effector protein, which was expressed from the high–copy number vector pESC-URA (Figure 2A) [23]. To ascertain the function of LI0666 in another genetic background, we transformed the pRS416-Gal1-3 × flag-LI0666 plasmid into strain BY4741. Comparable lethality was observed in BY4741 and W303-1A (Figure 2A). The activity of bacterial EPIYA effectors largely depends on tyrosine phosphorylation. To ascertain the contribution of tyrosine to the LI0666-mediated toxicity observed in the yeast system, we replaced the tyrosine residue within the LI0666 EPIYA site with an alanine residue and evaluated the lethality of the recombinant LI0666^Y149/184A^ variant. LI0666^Y149/184A^ was significantly less lethal to yeast than WT LI0666, underscoring the importance of the tyrosine residue in the functionality of LI0666 in a yeast system (Figure 2A).

Although *S. cerevisiae* lacks endogenous tyrosine kinases, it does express some dual-specificity Ser/Thr kinases that are also committed to tyrosine phosphorylation [24]. To investigate the level of LI0666 expression and tyrosine phosphorylation, yeast cell lysates were prepared and immunoblotted with an anti-FLAG and an anti-phosphotyrosine antibody. Our results show that, when expressed in yeast, WT LI0666 was stable and formed a tetramer (the molecular weight of the monomer is 24 kDa) or a large complex, while LI0666^Y149/184A^ did not polymerize and was unstable. Moreover, no tyrosine phosphorylation of LI0666 was detected (Figure 2B). These results suggest that the cytotoxicity of LI0666 in yeast is dependent on the tyrosine residue in the EPIYA motif, but not on tyrosine phosphorylation.

### LI0666 EPIYA motifs undergo tyrosine phosphorylation in mammalian cells

**Figure 3:**
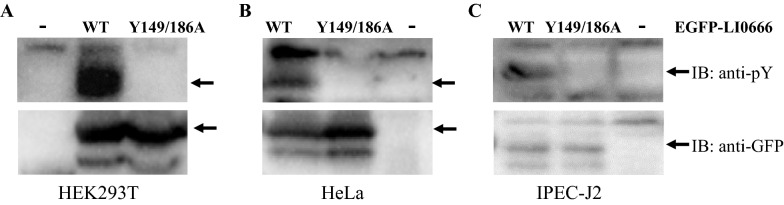
**LI0666 tyrosine phosphorylation in mammalian cells.** EGFP-LI0666 and EGFP-LI0666^Y149/186A^ were transfected into HEK293T (**A**), HeLa (**B**), and IPEC-J2 (**C**) cells. Cell lysates were subjected to Western blot using an anti-GFP antibody and a 4G10 antibody. Lysate from cells containing empty vector was used as the negative control.

To investigate whether LI0666 is tyrosine-phosphorylated at its EPIYA motifs in mammalian cells, constructs encoding EGFP-LI0666 and EGFP-LI0666^Y149/186A^ were transiently transfected into HEK293T cells, which were then immunoblotted with an anti-GFP antibody and a 4G10 antibody. As shown in Figure 3A, EGFP-LI0666 was expressed and efficiently tyrosine-phosphorylated in HEK293T cells, whereas EGFP-LI0666^Y149/184A^ was expressed but not phosphorylated. This phenotype was also observed in HeLa cells and in IPEC-J2 cells (the natural host for *L. intracellularis*) (Figures 3B and C). These results indicate that LI0666 undergoes tyrosine phosphorylation in porcine epithelial cell cytoplasm when delivered by a T3SS, and that this phosphorylation can also be carried out by kinases in human epithelial cells.

### EPIYA motifs are not required for membrane localization of LI0666 in *S. cerevisiae* or in mammalian cells

**Figure 4:**
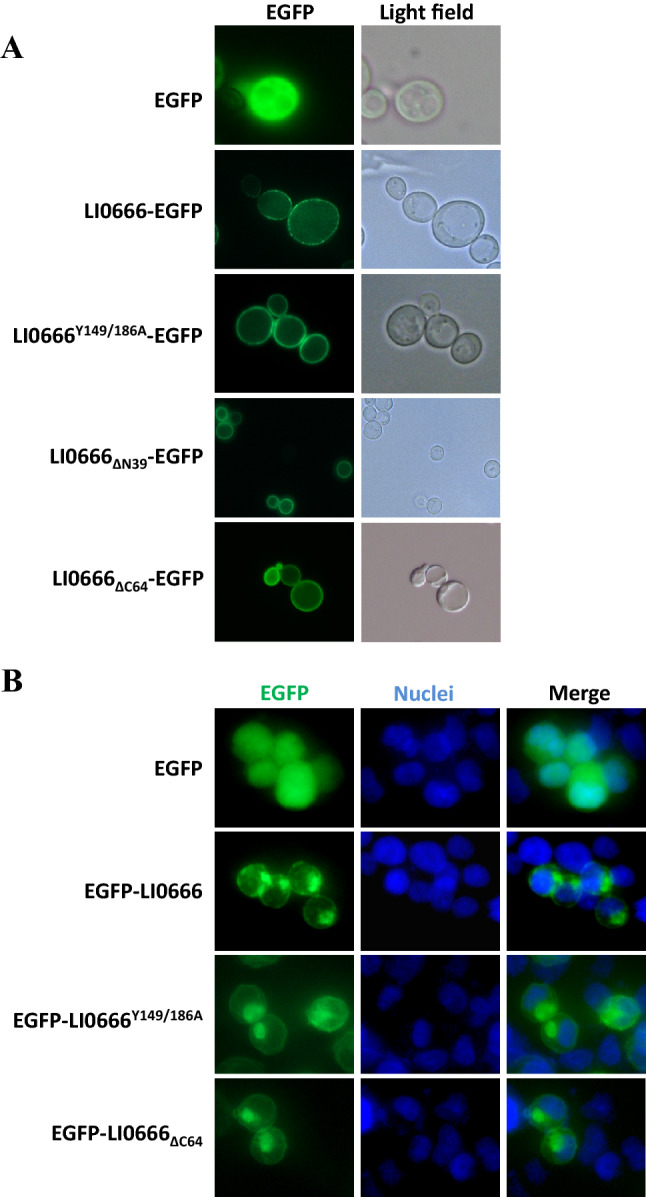
**Subcellular localization of GFP-LI0666 fusion proteins in *S. cerevisiae* and mammalian cells. A** Cells were cultured for 10 h under inducting conditions in galactose medium at 30 °C. Empty vector (pYES2/NTA encoding EGFP) was used as the negative control. LI0666-EGFP, LI0666^Y149/186A^-EGFP, LI0666_ΔN39_-EGFP and LI0666_ΔC64_-EGFP, which is a deletion mutant of LI0666 lacking the C-terminal 64 amino acids, was expressed in yeast respectively. **B** EGFP-LI0666, EGFP-LI0666^Y149/186A^ and EGFP-LI0666_ΔC64_ were transfected into HEK293T cells. After incubation at 37 °C for 24 h, the nuclei were stained with DAPI (4′,6-diamidino-2-phenylindole), and fluorescence was observed using a fluorescence microscope.

We next sought to determine the subcellular localization of LI0666 in yeast. To do this, we constructed a plasmid encoding EGFP fused to the C-terminus of LI0666 and transformed it into yeast. The tagged LI0666 protein localized to the plasma membrane (Figure 4A). The LI0666^Y149/184A^ mutant also localized to the plasma membrane, indicating that membrane localization of LI0666 is independent of the tyrosine residue within the EPIYA motifs. To identify the region of LI0666 that is responsible for membrane localization, we generated constructs expressing LI0666 mutants that lack the N-terminal region (LI0666_ΔN39_) or C-terminal region (harboring two EPIYA motifs)(LI0666_ΔC64_). LI0666_ΔN39_ and LI0666_ΔC64_ both localized to the plasma membrane, indicating that, like CagA, stable association of LI0666 with the membrane does not require the EPIYA-containing region (Figure 4A).

To further investigate the subcellular localization of LI0666 in mammalian cells, vectors expressing LI0666 fused to EGFP were transiently transfected into HEK293T cells. EGFP–LI0666 was localized to the plasma membrane, as well as to an intracellular component that appeared to be vesicular. This distribution pattern was similar to that observed for both LI0666^Y149/184A^ and EGFP-LI0666_ΔC64_, indicating that neither the tyrosine residue nor the EPIYA-containing region influences the intracellular distribution of LI0666 (Figure 4B). These results indicate that, like CagA, the membrane localization signal of LI0666 is located within the middle portion of the protein and is independent of the EPIYA-containing region.

## Discussion

In the present work, we demonstrate that *L. intracellularis* LI0666, a protein that contains two EPIYA motifs, appears to be a T3SS effector, because it is secreted by the *Y. enterocolitica* T3SS secretion system and inhibits yeast growth. LI0666 localized to the plasma membrane of yeast and mammalian cells, and neither EPIYA tyrosine phosphorylation nor the EPIYA-containing region were required for this localization pattern. Furthermore, LI0666 underwent tyrosine phosphorylation of the EPIYA motif in mammalian cells.

We previously reported that LI1035 inhibits yeast growth and is the first putative *L. intracellularis* effector [15]. In the current study, we found that LI0666 was exported by the *Yersinia* T3SS and also inhibited yeast growth, indicating that it is most likely an authentic effector protein. LI0666-mediated inhibition of yeast growth was not dependent on tyrosine phosphorylation, but did require the presence of the tyrosine residues in the EPIYA motifs, which may be related to their effect on protein polymerization or stability. We also expressed two other EPIYA-containing proteins, LI0041 and LIC053, in *S. cerevisiae*, and found that their expression did not alter the yeast growth (data not shown). Further experiments are needed to test these two proteins.

Previous reports indicated that the Domain II in the middle portion of CagA, but not the EPIYA containing region, is required for its membrane association [19, 20]. LI0666 was found to localized to the plasma membrane in yeast and mammalian cells in our experiment, which also independent of the EPIYA motif and the EPIYA-containing region. Therefore, elucidating the function of LI0666 may provide further insights into the mechanisms underlying the generation and evolution of bacterial EPIYA effectors during the coevolution of bacterial pathogens with their hosts.

Here we identified LI0666 as a new *L. intracellularis* EPIYA effector. Identification of the tyrosine kinase members responsible for LI0666 phosphorylation and the SH2 domain-containing host proteins that LI0666 interacts with will help to explore the molecular mechanisms of LI0666 in disease development.

## Data Availability

All data generated or analyzed during this study are included in this published article.
